# Predictive model for urosepsis in patients with Upper Urinary Tract Calculi based on ultrasonography and urinalysis using artificial intelligence learning

**DOI:** 10.1590/S1677-5538.IBJU.2022.0450

**Published:** 2022-12-15

**Authors:** Xuwei Hong, Guoyuan Liu, Zepai Chi, Tenghao Yang, Yonghai Zhang

**Affiliations:** 1 Department of Urology Sun Yat-sen Memorial Hospital Sun Yat-sen University Guangzhou China Department of Urology, Sun Yat-sen Memorial Hospital, Sun Yat-sen University, Guangzhou, P. R. China;; 2 Department of Urology Shantou Central Hospital Shantou China Department of Urology, Shantou Central Hospital, Shantou, 515031, P. R. China

**Keywords:** Urinary Calculi, Ultrasonography, Neural Networks, Computer

## Abstract

**Purpose:**

To construct a predicting model for urosepsis risk for patients with upper urinary tract calculi based on ultrasound and urinalysis.

**Materials and Methods:**

A retrospective study was conducted in patients with upper urinary tract calculi admitted between January 2016 and January 2020. The patients were randomly grouped into the training and validation sets. The training set was used to identify the urosepsis risk factors and construct a risk prediction model based on ultrasound and urinalysis. The validation set was used to test the performance of the artificial neural network (ANN).

**Results:**

Ultimately, 1716 patients (10.8% cases and 89.2% control) were included. Eight variables were selected for the model: sex, age, body temperature, diabetes history, urine leukocytes, urine nitrite, urine glucose, and degree of hydronephrosis. The area under the receiver operating curve in the validation and training sets was 0.945 (95% CI: 0.903-0.988) and 0.992 (95% CI: 0.988-0.997), respectively. Sensitivity, specificity, and Yuden index of the validation set (training set) were 80.4% (85.9%), 98.2% (99.0%), and 0.786 (0.849), respectively.

**Conclusions:**

A preliminary screening model for urosepsis based on ultrasound and urinalysis was constructed using ANN. The model could provide risk assessments for urosepsis in patients with upper urinary tract calculi.

## INTRODUCTION

Urosepsis is a life-threatening organ dysfunction caused by the dysregulated host response to infection originating from the urinary tract and/or male genital organs ( 1 ). The latest definition states that urosepsis is more severe than an uncomplicated urinary infection, implying the need for prompt recognition and intervention ( 2 ). Urosepsis must be diagnosed early and treated promptly to prevent progression to septic shock and multiple organ dysfunction ( 3 , 4 ). Upper urinary tract obstruction caused by calculi is an important cause of urosepsis ( 5 ). Currently, most of the studies focus on the risk factors of urosepsis following endoscopic lithotripsy ( 6 - 8 ). However, in the clinic, many patients are diagnosed with upper urinary tract calculi complicated with urosepsis before or after admission ( 9 ). Therefore, the early identification of high-risk upper urinary tract calculi patients at risk of developing urosepsis and the implementation of effective intervention methods have become a priority recognized by the World Health Organization ( 2 , 10 ).

Ultrasound is a common emergency imaging technique in patients presenting severe loin pain and fever. It can reveal the size, location, and degree of obstruction of urinary calculi and also help evaluate complications of acute pyelonephritis, such as renal abscess, emphysematous pyelonephritis, and perirenal abscess ( 11 , 12 ). Urinalysis, including the assessment of white and red blood cells and nitrite, can reflect the urinary inflammatory response quickly. It is recommended as a routine detection and suggested for repetitive analysis. In addition, urine culture and antimicrobial susceptibility testing must be performed in all cases of pyelonephritis ( 13 ).

Nowadays, artificial intelligence is commonly used in disease diagnosis, treatment, and prognosis prediction ( 14 , 15 ). Artificial neural network (ANN) is the most popular method for machine learning. It is a kind of non-parametric modeling technique, which is suitable for complex phenomenon that investigators do not know underlying functions. ANN is in analogue to the human brain. There are input and output signals transmitting from input to output nodes. Input signals are weighted before reaching output nodes according to their respective importance. Then the combined signal is processed by activation function. ANN has better predictive performance and can grasp the inherent data patterns more effectively than traditional statistical methods ( 16 , 17 ). It has been applied widely in urological practice, including distinction between tumor grade or subtype of genitourinary malignancies, prediction of treatment response, tumor recurrence, and patient survival. The most common ANN application in urolithiasis is in the prediction of endourologic surgical outcomes and stone-free status after Extracorporeal Shock Wave Lithotripsy ( 18 , 19 ). Currently, no studies using ANN data mining approach to explore the risk of upper urinary tract calculi complicated with urosepsis are available.

This study aimed to construct a urosepsis risk prediction model based on ultrasound and urinalysis for patients with upper urinary tract calculi using the ANN data mining approach. This model can be used as a preliminary screening tool to identify patients who are at high risk of urosepsis and be helpful in guiding targeted examinations or interventions.

## MATERIALS AND METHODS

### Study design and population

This retrospective study included patients with upper urinary tract calculi admitted to Shantou Central Hospital between January 2016 and January 2020. The inclusion criteria were 1) imaging results, including urinary system ultrasound, excretory urogram, or abdominopelvic computed tomography (CT) indicating a diagnosis of ureteral calculi, and 2) complete medical history, laboratory, and imaging data available. The exclusion criteria were 1) <14 years of age or pregnancy (women), 2) bilateral upper urinary calculi, 3) diseases of the blood or immune system, malignancy, or use of immunoregulatory therapy, or 4) other sites of primary infection, including lung or abdomen. Urosepsis was diagnosed based on the guidelines for diagnosis and treatment of urosepsis in 2018 ( 2 ).

The study followed the Declaration of Helsinki and was approved by the ethics committee for medical research at Shantou Central Hospital (IRB Number: 2019-sci-No.070). Due to the retrospective nature of the study, the requirement for informed consent was waived by the ethics committee.

### Data collection

Data including sex, age, body temperature, abdominal pain, hematuria, urinary irritation symptoms, hypertension, diabetes, calculi surgery history, urine leukocytes (U-LEU), urine nitrite (U-NIT), urine erythrocytes (U-ERY), urine glucose (U-GLU), laterality of calculi, location of calculi, degree of hydronephrosis, and the maximal diameters of calculi were collected from medical records. Urinalysis was performed on a Mindray (UA-5800) automatic dry chemical urine analyzer and matching test strips. Ultrasound analysis was performed on a Hitachi (EUB 5500) full digital color Doppler ultrasound diagnostic system.

### Sample sets for ANN development and validation

**Figure 1:**
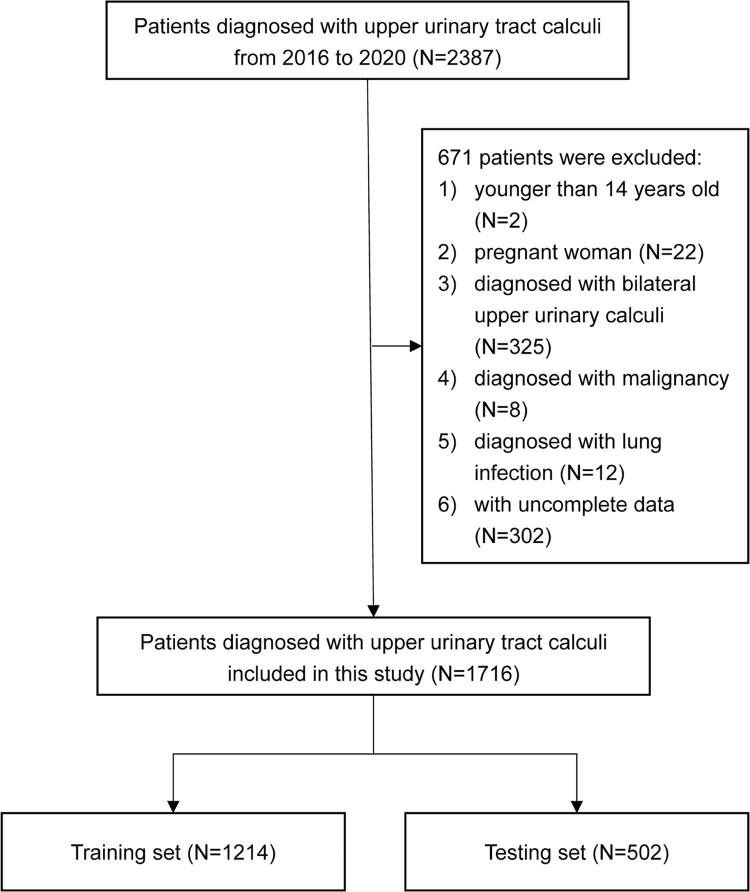
Flow diagram of the selection of eligible A

For ANN model construction and validation, the patients were randomized into the training (1214 patients; 135 cases and 1079 controls) and validation (502 patients; 51 cases and 451 controls) sets ( Figure-1 ). Randomization was performed using SPSS 25.0 (IBM, Armonk, NY, USA).

### Selection of the variables for ANN model development

Univariable and multivariable logistic regression analyses were performed to evaluate variables associated with urosepsis and generate the ANN model for the training set. Variables with p<0.05 were selected for predictive model establishment.

### Development of the ANN model

A standard feed-forward backpropagation neural network (BPNN) was applied, consisting of three layers: an input layer that receives information, a hidden layer that processes information, and an output layer that calculates results. BPNN was run with significant predictors as input variables and urosepsis risk as the output variable. The number of neurons in the input layer was the total number of covariables. The output variable was dichotomous (two neurons in the output layer). The number of neurons in the hidden layer was not an actual variable. The optimal H was determined by trial and error since no authoritative theory is available. The optimal hidden layer was determined from the predictive model with the highest sensitivity and specificity. In BPNN, the variables of the upper layer were weighted and related to the next layer by transfer functions. In the constructed BPNN, hyperbolic tangent functions were used as the transfer functions of the hidden layers, and SoftMax functions were used as transfer functions for the output layers. Training parameters, including learning rate and momentum, were set at the default values. The networks were trained at a maximum of 100 epochs or until the minimum average square error was <0.001.

### Validation of the ANN model

The accuracy, positive (PPR) and negative (NPR) predictive rates, sensitivity, specificity, Youden Index, and area under the receiver operating characteristics (ROC) curve (AUC) were determined in both sets. The Hosmer-Lemeshow goodness-of-fit test was performed for ANN model calibration (p>0.05).

## Statistical analysis

Normally distributed continuous variables were presented as means ± standard deviation (SD). Categorical were presented as numbers and proportions. Student’s t-test and Pearson chi-square test were used to analyze continuous and categorical variables, respectively. SPSS 25.0 (IBM, USA) was used for data analysis. Two-tailed p<0.05 was considered statistically significant.

## RESULTS

Initially, 2387 patients were screened, and 1716 were included. There were 186 (10.8%) patients with urosepsis (cases) and 1530 (89.2%) without (controls). In both sets, the proportion of males was 56.8%. In the training set, 186 (15.3%) patients had diabetes, 302 (24.9%) had hypertension, and 165 (13.6%) underwent calculi surgery, while in the validation set, there were 62 (12.4%), 127 (25.3%), and 66 (13.1%) patients, respectively ( Table-1 ).

**Table 1:** Baseline characteristics of the patients with upper urinary tract calculi in the training and validation sets.

Characteristics	Training set (n=1214)	Validation set (n=502)	𝒳^2^/t	p
**Urosepsis (n, %)**			0.339	0.560
Yes	135 (11.1%)	51 (10.2%)		
No	1079 (88.9%)	451 (89.8%)		
**Sex (n, %)**			0	0.994
Male	689 (56.8%)	285 (56.8%)		
Female	525 (43.2%)	217 (43.2%)		
Age (yeas old, std)	52.7(12.3)	52.3(12.4)	0.620	0.535
**Body temperature (n, %)**			0.441	0.802
Normal	1055 (86.9%)	442 (88.0%)		
High	153 (12.6%)	58 (11.6%)		
Low	6 (0.5%)	2(0.4%)		
**Abdominal pain (n, %)**			0.666	0.414
Yes	559 (46.0%)	242 (48.2%)		
No	655 (54.0%)	260 (51.8%)		
Hematuria (n, %)			2.651	0.103
Yes	221 (18.2%)	75 (14.9%)		
No	993 (81.8%)	427 (85.1%)		
**Urinary irritation symptoms (n, %)**			2.073	0.150
Yes	571 (47.0%)	217 (43.2%)		
No	643 (53.0%)	285 (56.8%)		
Diabetes (n, %)			2.535	0.111
Yes	186 (15.3%)	62 (12.4%)		
No	1028 (84.7%)	440 (87.6%)		
**Hypertension (n, %)**			0.034	0.854
Yes	302 (24.9%)	127 (25.3%)		
No	912 (75.1%)	375 (74.7%)		
**Treatment history (n, %)**			0.060	0.806
Yes	165 (13.6%)	66 (13.1%)		
No	1049 (86.4%)	436 (86.9%)		
**U-LEU (n, %)**			5.620	0.132
(-)	364 (30.0%)	155 (30.9%)		
(1+)	338 (27.8%)	113 (22.5%)		
(2+)	220 (18.1%)	103 (20.5%)		
(3+)	292 (24.1%)	131 (26.1%)		
**U-NIT (n, %)**			0.060	0.806
(+)	165 (13.6%)	66 (13.1%)		
(-)	1049 (86.4%)	436 (86.9%)		
**U-ERY (n, %)**			3.492	0.322
(-)	341 (28.1%)	135 (26.9%)		
(1+)	304 (25.0%)	127 (25.3%)		
(2+)	269 (22.2%)	130 (25.9%)		
(3+)	300 (24.7%)	110 (21.9%)		
**U-GLU (n, %)**			4.797	0.187
(-)	1044 (86.0%)	448 (89.2%)		
(1+)	49 (4.0%)	15 (3.0%)		
(2+)	94 (7.7%)	34 (6.8%)		
(3+)	27 (2.2%)	5 (1.0%)		
**Laterality of calculi (n, %)**			0.928	0.335
Right	609 (50.2%)	239 (47.6%)		
Left	605 (49.8%)	263 (52.4%)		
**Location of calculi (n, %)**			0.529	0.467
Ureter	968 (79.7%)	408 (81.3%)		
Kidney	246 (20.3%)	94 (18.7%)		
Max-diameter of calculi (mm, std)	18.9 (9.2)	18.2 (8.5)	1.575	0.116
**Degree of hydronephrosis (n, %)**			3.061	0.382
No	199 (16.4%)	71 (14.1%)		
Mild	364 (30.0%)	152 (30.3%)		
Moderate	346 (28.5%)	161 (32.1%)		
Severe	305 (25.1%)	118 (23.5%)		

U-LEU = urine leukocytes; U-NIT = urine nitrite; U-ERY = urine erythrocytes; U-GLU = urine glucose.

The input variables in the predictive model included sex, age, body temperature, diabetes history, U-LEU, U-NIT, U-GLU, and degree of hydronephrosis. The multivariable analysis showed that old age (OR=1.055, 95%CI: 1.030-1.08), abnormal body temperature (high vs. normal, OR=7.636, 95%CI: 4.102-14.216; low vs. normal, OR=85.545, 95%CI: 3.316-2206.854), positive U-LEU (1+ vs. negative, OR=4.250, 95%CI: 1.336-13.518; 2+ vs. negative, OR=6.452, 95%CI: 2.050-20.308; 3+ vs. negative, OR=10.092, 95%CI: 3.416-29.818), positive U-NIT (positive vs. negative, OR=6.173, 95%CI: 3.409-11.178), positive U-GLU (2+ vs. negative, OR=5.639, 95%CI: 1.609-19.771; 3+ vs. negative, OR=14.255, 95%CI: 2.652-76.630), and mild and moderate degree of hydronephrosis (mild vs. no, OR=3.793, 95%CI: 1.577-9.124; moderate vs. no, OR=2.488, 95%CI: 1.018-6.081) were independent risk factors of urosepsis for upper urinary calculi patients ( Table-2 ).

**Table 2:** Univariable and multivariable logistic regression analyses for the development of urosepsis in the training set of patients with upper urinary tract calculi.

	Univariable analysis		Multivariable analysis	

	OR (95%CI)	P	OR (95%CI)	P
**Sex**				
Male vs. female	0.592 (0.413-0.848)	0.004	1.004 (0.594-1.698)	0.987
**Age**				
Continuous	1.055 (1.038-1.073)	<0.001	1.055 (1.030-1.080)	<0.001
**Body temperature**				
High vs. normal	33.830 (21.637-52.893)	<0.001	7.636 (4.102-14.216)	<0.001
Low vs. normal	123.537 (14.111-1081.554)	<0.001	85.545 (3.316-2206.854)	0.007
**Abdominal pain**				
Yes vs. no	1.096 (0.764-1.572)	0.619		
**Hematuria**				
Yes vs. no	1.395 (0.907-2.145)	0.13		
**Urinary irritation symptoms**				
Yes vs. no	1.374 (0.959-1.967)	0.083		
**Diabetes**				
Yes vs. no	9.299 (6.301-13.725)	<0.001	0.452 (0.135-1.514)	0.198
**Hypertension**				
Yes vs. no	1.367 (0.923-2.024)	0.118		
**Treatment history**				
Yes vs. no	1.524 (0.954-2.437)	0.078		
**U-LEU**				
(1+) vs. (-)	4.999 (1.871-13.355)	0.001	4.250 (1.336-13.518)	0.014
(2+) vs. (-)	11.777 (4.505-30.783)	<0.001	6.452 (2.050-20.308)	0.001
(3+) vs. (-)	25.714 (10.246-64.537)	<0.001	10.092 (3.416-29.818)	<0.001
**U-NIT**				
(+) vs. (-)	22.216 (14.612-33.778)	<0.001	6.173 (3.409-11.178)	<0.001
**U-ERY**				
(1+) vs. (-)	0.933 (0.563-1.545)	0.788		
(2+) vs. (-)	1.352 (0.833-2.193)	0.222		
(3+) vs. (-)	0.879 (0.526-1.468)	0.623		
**U-GLU**				
(1+) vs. (-)	2.845 (1.282-6.313)	0.01	1.154 (0.302-4.410)	0.834
(2+) vs. (-)	15.216 (9.471-24.447)	<0.001	5.639 (1.609-19.771)	0.007
(3+) vs. (-)	11.666 (5.250-25.922)	<0.001	14.255 (2.652-76.630)	0.002
**Laterality of calculi**				
Left vs. right	1.059 (0.740-1.515)	0.753		
**Location of calculi**				
Ureter vs. kidney	0.796 (0.521-1.217)	0.292		
**Max-diameter of calculi**				
Continuous	1.011 (0.993-1.030)	0.239		
**Degree of hydronephrosis**				
Mild vs. no	3.710 (1.958-7.031)	<0.001	3.793 (1.577-9.124)	0.003
Moderate vs. no	2.450 (1.266-4.738)	0.008	2.488 (1.018-6.081)	0.046
Severe vs. no	0.313 (0.115-0.847)	0.022	0.201 (0.055-0.728)	0.015

U-LEU = urine leukocytes; U-NIT = urine nitrite; U-ERY = urine erythrocytes; U-GLU = urine glucose.

**Figure 2:**
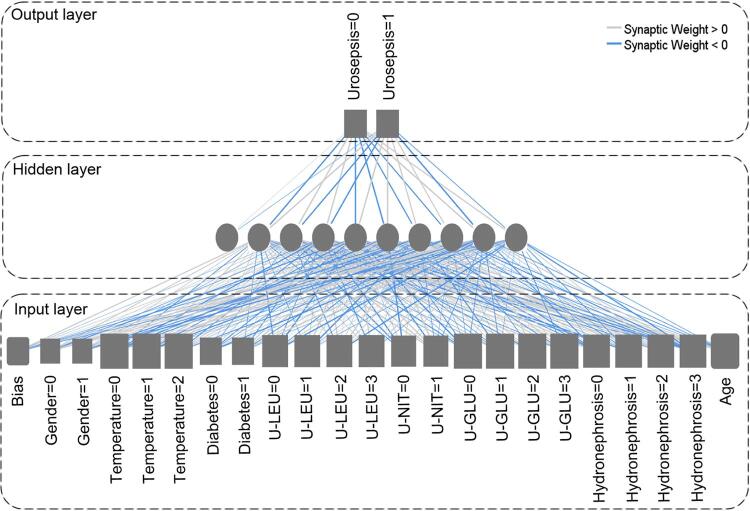
Artificial neural network for predicting urosepsis in patients with upper urinary tract calculi. The gray boxes and circles represent neurons, and the lines between boxes and circles represent modifiable connections. For urosepsis, 0 and 1 present no and yes, respectively; for gender, 0 and 1 present female and male, respectively; for temperature, 0, 1 and 2 present normal, and high and low, respectively; for diabetes, 0 and 1 present no and yes, respectively; for U-LEU, U-NIT, and U-GLU, 0, 1, 2 and 3 present(-), (+), (2+) and (3+), respectively; for hydronephrosis, 0, 1, 2 and 3 present no, mild, moderate and severe, respectively.

An ANN model was built based on the significantly associated variables. The input variables were the eight significant variables mentioned above, and the output variable was dichotomous (urosepsis or not). The ANN model consisted of an input layer, a hidden layer, and an output layer. The input and output layers contained 22 and two neurons, depending on the number of input and output variables, respectively. The number of neurons in the hidden layer was calculated automatically according to the model’s architecture, including the number of hidden layers and the activation function of the hidden layer and output layer. Each neuron in the different layers was connected by a mathematical function that simulates synapses. Finally, a 3-layer BPNN model with 22, nine, and two neurons in the input, hidden, and output layers, respectively, was constructed as the best predictive model ( Figure-2 ).

**Figure 3:**
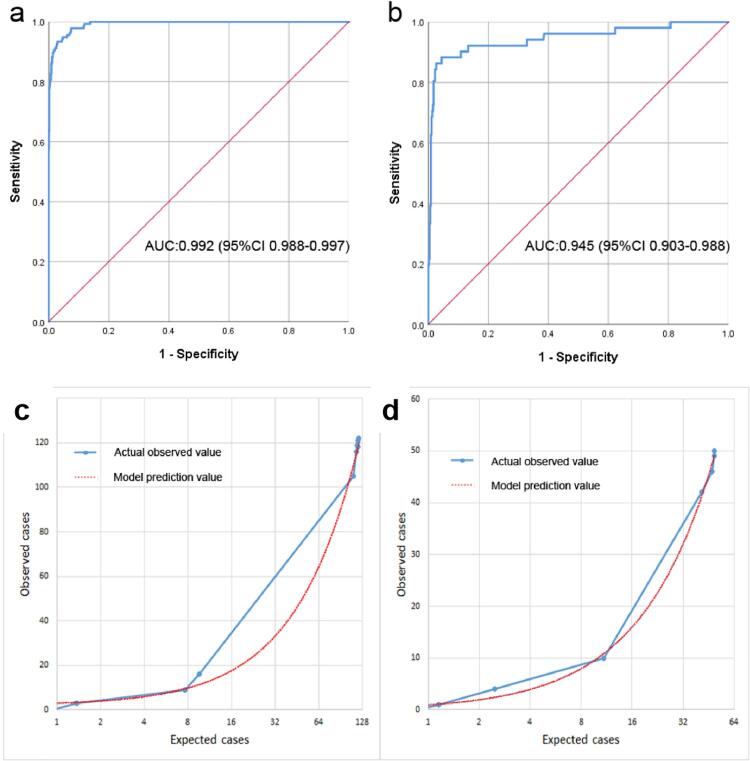
ROC curve and calibration of the nomogram for predicting urosepsis for upper urinary tract calculi patients. (a) ROC curve in the training set; (b) ROC curve in the validation set. Calibration curve of the ANN model for the training set (c) and the validation set (d).

The ROC AUC was used to validate the ANN model. The AUCs of the training ( Figure-3a ) and validation ( Figure-3b ) sets were 0.992 (95% CI: 0.988-0.997) and 0.945 (95% CI: 0.903-0.988), respectively. The accuracies of the training and validation sets were 97.5% and 96.4%, respectively. The PPR and NPR of the validation set (training set) were 83.7% (91.3%) and 97.8% (98.3%), respectively. The sensitivity, specificity, and Youden Index of the validation set (training set) were 80.4% (85.9%), 98.2% (99.0%), and 0.786 (0.849), respectively.

The ANN model was calibrated using the Hosmer-Lemeshow goodness-of-fit test and calibration plot. The Hosmer-Lemeshow test revealed high concordance between the predicted and observed probabilities for the training (p=0.093) and validation (p=0.868) sets. The calibration plot also showed good agreement between the predicted and observed outcomes for the training ( Figure-3c ) and validation ( Figure-3d ) sets.

## DISCUSSION

The present study developed a prediction model for urosepsis using ANN, involving eight significant predictors, including sex, age, diabetes history, body temperature, U-LEU, U-NIT, U-GLU, and degree of hydronephrosis. The ANN model showed encouraging outcomes regarding its ability in the early identification of urosepsis in patients with upper urinary tract calculi urosepsis based on ultrasound and urinalysis. The prediction model could be a rapid, clinically applicable risk assessment method to predict urosepsis in patients with upper urinary tract calculi.

Recent studies consistently found the superiority of the ANN analysis over traditional statistical methods ( 18 ). In this study, the ANN model was proved to have a better performance compared to the Nomogram model, which was used to predict probability of patients with ureteral calculi developing into urosepsis in a previous study ( 9 ). The AUC values of the ANN model and Nomogram model in the training (validation) groups were 0.992 (0.945) and 0.914 (0.874) respectively. Compared to conventional regression methods, ANN did not require a predefined mathematical relationship between the dependent and independent variables, and could model any arbitrarily complicated nonlinear relationship ( 20 ). Theoretically, the ANN model could be built more accurately and perfectly by increasing the sample size and repeated training. These advantages enable ANN to be a useful tool in solving the complex challenge of prediction.

Few clinical studies assessed the probability of patients with upper urinary calculi developing urosepsis ( 9 , 21 ). The risk factors for upper urinary tract calculi complicated by urosepsis remain unclear. In this study, we revealed age, fever, urinary white blood cells, urinary nitrite, urinary glucose, and hydronephrosis were independent risk factors for urosepsis in upper urinary calculi patients. Aging is often accompanied by liver, kidney, cardiovascular, and immune system dysfunctions. Older patients often have comorbidities, including hypertension and diabetes. Once ureteral obstruction occurs, they are prone to secondary infections and progress to systemic inflammatory response syndrome and even sepsis ( 22 , 23 ). This study suggested that body temperature alterations in patients with upper urinary tract calculi could also independently predict urosepsis. Fever occurs in response to endogenous and/or exogenous pyrogenic substances, including lipopolysaccharide (LPS) produced by Gram-negative bacteria ( 24 ). Most patients with sepsis have a fever, while only 10%-29% of the patients are hypothermic, showing even higher disease severity and mortality rate ( 25 ).

Consistent with previous studies ( 9 , 26 ), two infection-related indicators in urinalysis, U-LEU and U-NIT, were confirmed as independent risk factors for urosepsis in patients with upper urinary tract calculi. Positive urine culture is also associated with urosepsis ( 7 , 8 ). However, in this study, urine culture was not selected as a candidate risk factor due to its hysteresis characteristic. In the clinic, urine culture often takes 2-3 days or more to produce results, which is inconsistent with the purpose of this study to identify high-risk patients with urosepsis as soon as possible. Positive U-LEU often indicates purulent inflammation of the urinary tract, whose commonest cause is bacterial infections. In addition, Gram-negative bacilli in the urinary tract reduce nitrate, a protein metabolite in urine, to nitrite. Therefore, U-LEU and U-NIT detection can quickly and indirectly determine the possibility of bacterial infection in the urinary system ( 27 , 28 ).

The common causes of U-GLU positivity include elevated blood glucose and decreased renal glucose threshold. When blood glucose rises and exceeds the upper limit of renal tubular reabsorption, glucose is excreted in the urine, resulting in positive U-GLU. In addition, some kidney diseases also decrease the ability of renal tubules to reabsorb glucose. In this case, even if the blood glucose is normal, U-GLU positivity occurs ( 29 ). This study found that U-GLU 2+ and 3+ were risk factors for urosepsis. Positive U-GLU of 2+ or 3+ indicates poor control of diabetes or the possibility of chronic kidney disease. Once a patient suffers from urinary tract infection, the risk of developing into urosepsis is higher.

Among ultrasound-related indicators, only the degree of hydronephrosis independently predicted urosepsis. Hydronephrosis mainly indicates urinary tract obstruction. Once the patients have urinary tract infections, bacteria in the urine retrograde into the blood after reaching a certain pressure, resulting in urosepsis ( 30 ). Interestingly, this study showed that severe hydronephrosis was negatively correlated with urosepsis risk. Urosepsis commonly appears as an acute course. Severe hydronephrosis indicates a tight and prolonged obstruction, which makes it difficult for bacteria to cause retrograde infection.

This study had limitations. Firstly, it was a retrospective observational study with unavoidable selection bias. However, strict eligibility criteria were adopted. In addition, we randomly composed the training and validation sets to minimize selection bias. Secondly, the training and validation sets were from the same population, so the model might not be generalizable. Therefore, large multicenter studies are needed. Thirdly, the ANN model was based on general information, symptoms, ultrasound, and urinalysis. Data collection, especially for symptoms, was based on self-reports, with inevitable recall bias. Lastly, certain populations were excluded, e.g., patients with bilateral upper urinary calculi. In this study, the inclusion of patients with bilateral upper urinary calculi would lead to difficult grouping, and some indicators could not be well grouped. In addition, the study also excluded people with malignant tumors or immune system diseases because these patients have more interference factors. Although these populations were unsuitable for this study model, they were also those we need to focus on clinically.

## CONCLUSIONS

Despite the limitations, this is the first study using ANN to estimate the urosepsis risk for upper urinary tract calculi base on ultrasound and urinalysis. This model could help determine the probability of urosepsis and then perform targeted examinations or interventions, which would be more efficient to improve the efficiency of diagnosis and treatment.
